# Five decades on: Use of historical weaning size data reveals that a decrease in maternal foraging success underpins the long-term decline in population of southern elephant seals (*Mirounga leonina*)

**DOI:** 10.1371/journal.pone.0173427

**Published:** 2017-03-16

**Authors:** Ella Clausius, Clive R. McMahon, Mark A. Hindell

**Affiliations:** 1 Centre for Ecology and Biodiversity, Institute for Marine and Antarctic Studies, University of Tasmania, Hobart, Tasmania, Australia; 2 Sydney Institute of Marine Science, Mosman, New South Wales, Australia; 3 Antarctic Climate and Ecosystems CRC, University of Tasmania, Hobart, Tasmania, Australia; Phillip Island Nature Parks, AUSTRALIA

## Abstract

The population of Southern elephant seals (*Mirounga leonina*) at Macquarie Island has declined since the 1960s, and is thought to be due to changing oceanic conditions leading to reductions in the foraging success of Macquarie Island breeding females. To test this hypothesis, we used a 55-year-old data set on weaning size of southern elephant seals to quantify a decrease in weaning size from a period of population stability in 1950s to its present state of on-going decline. Being capital breeders, the size of elephant seal pups at weaning is a direct consequence of maternal foraging success in the preceding year. During the 1940-1950s, the mean of female pups at weaning was similar between the Heard and Macquarie Island populations, while the snout-tail-length length of male weaners from Heard Island were longer than their conspecifics at Macquarie Island. Additionally, the snout-tail-length of pups at weaning decreased by 3cm between the 1950s and 1990s in the Macquarie Island population, concurrent with the observed population decline. Given the importance of weaning size in determining first-year survival and recruitment rates, the decline in the size at weaning suggests that the decline in the Macquarie Island population has, to some extent, been driven by reduced maternal foraging success, consequent declines in the size of pups at weaning, leading to reduced first-year survival rates and recruitment of breeding females into the population 3 to 4 years later.

## Introduction

The body of work quantifying the effects of climate change on animal populations has grown rapidly, and encompasses most, if not all, major taxonomic groups across all the World’s oceans and continents [1–4]. This work is only made possible by long-term datasets covering a range of both environmental and biological conditions [5]. Thus, for species or regions in which the collection of long-term and continuous data is difficult, identifying the relationships between environmental change and changes in the dynamics and demographics of populations can be especially challenging. In the remote Antarctic and Southern Ocean adverse conditions hamper the collection of long-term biological data. As such, information on the size and trends of animal populations in the region is rare, with little to no information available prior to the 1950s, and only very scarce information available prior to the mid-1970s [6, 7].

The southern elephant seal (*Mirounga leonina)*, a wide-ranging and dominant predator in the Antarctic and Southern Ocean ecosystem, experienced major population declines across much of its circumpolar distribution throughout the latter half of the 20^th^ century [8]. While the major populations at Iles Kerguelen and Heard Island, Peninsula Valdes and South Georgia have stabilized or increased [9], the Macquarie Island population in the southern Pacific Ocean has declined continuously at a mean rate of 0.8% *per annum* since the 1950s [10]. Although the cause of this decline remains unknown, it is thought to be due to changing oceanic conditions leading to reductions in the foraging success of Macquarie Island breeding females through alterations in the availability or quality of their prey.

While it is often difficult to identify temporal changes in the quality or quantity of prey of wide-ranging and migratory marine predators, the size of southern elephant seals at weaning can be used as a broad-scale index representing relative changes in the foraging conditions encountered by breeding females during their *pre-partum* foraging migrations [11]. Since southern elephant seals are capital breeders fasting for the duration of the nursing period and raising their pup exclusively off stored energetic reserves [12], the foraging success of females over the *pre-partum* period (a function of foraging conditions over the winter months) strongly influences the energy expended on their pups over the lactation period and consequently the size of pups at weaning [13]. Further, the size of pups at weaning determines first-year survival rates [14] and ultimately the recruitment of females into the breeding population 3–4 years later [15]. Any changes in foraging conditions that affect female foraging success and the size of her pup at weaning can have long-term and potentially adverse implications for the growth and dynamics of the population [16].

If long-term declines in the foraging conditions encountered by breeding females are responsible for the population decline at Macquarie Island since the 1950s [17], we would expect to see a reduction in maternal foraging success in the present population compared to the 1950s before the decline started. This should be reflected in a reduction in the size of their pups at weaning over the same period.

Although the mass of pups at weaning is predominately used to reflect maternal foraging success, no long-term datasets of weaning mass exist for Macquarie Island. A long-term dataset of the snout tail length (*STL*) of pups at weaning is, however, available and covers a period of both population stability (1940s-1960s) and population decline (1990s). Because much of the growth of elephant seal pups during the short lactation period is due to the deposition of fat necessary to sustain them for the 6-week post-weaning fast, it is uncertain whether the *STL* of pups at weaning is similarly affected by maternal foraging success as weaning mass.

The primary aim of this study is therefore to test the hypothesis that the size of their pups at weaning was lower in the 1990s when the population was in decline compared to the 1950s when the population was stable. Specifically, we: (1) establish whether pup *STL* at weaning is a reliable measure of maternal expenditure (and therefore foraging success) by studying the relationship between growth in *STL* and mass over the lactation period; and, (2) identify spatial and temporal differences in maternal foraging success by comparing the weaning *STL* of pups from Heard Island in the Southern Indian Ocean and Macquarie Island in the southern Pacific Ocean during the mid-20^th^ century, and comparing the weaning *STL* of Macquarie Island pups from a period of population stability (1940-1960s) to those from a period of population decline (1990s).

## Methods

### Measurements of pups at birth and weaning

The data used in this study were collected from Heard Island (53°06’S, 73°31’E) and the Isthmus area of Macquarie Island (54°30’S, 158°57’E) [18] over 23 breeding seasons (September to November) between 1949 and 2005. These islands lie in different Oceans (the Southern Indian and Southern Pacific, and are 5240 km apart. The data were from: (i) Heard Is: 1949–1953, (ii) Macquarie Island; 1954–1963 and (iii) Macquarie Island: 1987–2005. The contemporary data were collected under permits from the Australian Antarctic Animal Ethics Committee (AAS 2265 & AAS 2794) and the Tasmanian Parks and Wildlife Service.

The size at weaning data from Heard and Macquarie Island can be divided into main sampling periods:

### Historical data (1949–1962)

The historical data were obtained the Australian Antarctic Data Centre under the entry ID: AADC-00102. Weaned elephant seal pups at Heard and Macquarie Island were measured when branded at 4-10-weeks using snout tail length (STL) to the nearest 6 inches (15.24 cm), either by eye, by pacing, by measuring cane or more accurately by marking the ground level with the nose and tail and measuring the distance [18, 19].

### Contemporary data (1987–2005)

Three weeks after the birth of the first pups each season, daily searches were conducted of the beach and tussock areas for recently weaned pups. Pups were presumed to have weaned when observed first outside of their natal harems, despite the presence of their mother in the harem at the time. New weaners were captured on the day of weaning, sexed and *STL* measured to the nearest 0.01m using a fiberglass tape measure [20–22]. The effect of collecting the length data at different ages for the historical and contemporary data set does not confound the analysis, because once weaned the pups no longer grow, in fact they lose weight over this time [23]. As there is no energy input there can be no somatic growth.

A total of 8494 individual elephant seal weaners were captured, sexed and measured over the two study periods. Mean sample size of all years was 360.30 ± 360.40 and sample size ranged from 1 to 1266. All sample sizes were above 30 except in 1993 (n = 2), 1994 (n = 1) and 1997 (n = 1).

### Growth in STL & mass over lactation

During the 1987, 1988 and 1996 breeding seasons, a subset of these pups (n = 24) were initially captured within 24 hours of birth, *STL* measured and weighed to the nearest kilogram using a 200kg dial face spring balance (Salter) suspended from an aluminum pole. These pups were tagged with two uniquely numbered plastic tags (Dalton Supplies, Ltd) in the interdigital webbing of their hind flippers to allow for their identification at weaning [24]. The birth and weaning data on these pups were used to quantify the growth in *STL* and mass over the lactation period. Changes in *STL* and mass were calculated by subtracting weaning *STL* from birth *STL* (*stl*.*growth*) and by subtracting weaning mass from birth mass (*m*.*growth*), respectively. A simple linear regression was conducted to determine the relationship between *stl*.*growth* and *m*.*growth*. The relationship between the change in *STL* and the change in mass over the lactation period was used to determine whether *STL* at weaning can provide a reliable proxy of weaning mass, and thus whether weaning *STL* accurately reflects maternal foraging performance.

### Statistical analysis

All statistical analyses were conducted in the statistics program R (version 3.1.2, R Core Team, 2016). Prior to conducting analyses all individuals with unknown sex (n = 207), one outlying record (274.3cm, Heard Island, 1949) and three years with small sample sizes from Macquarie Island (1993, n = 2; 1994, n = 1; 1997, n = 1) were removed. The Macquarie Island 1952 weaning length data were also excluded due to an exceptionally high mean length in this year (150.95 ± 9.85; n = 198) compared to the mean *length* for all sampled years (136.89 ± 6.59). This difference of 14cm is consistent with the measurement being snout-flipper length rather than snout-tail length.

### STL at weaning

Linear mixed-effects models (LMMs) were fitted to the weaning STL data from Heard and Macquarie Island using the *nlme* package [25] to explore the effects of sampling *PERIOD* and *SEX* on weaning *STL*. *YEAR* was included in these models as a random term, which accounted for any natural stochastic variation in mean weaning STL between years. Model parameters were fitted using Maximum Likelihood (ML) estimation. Model selection was based on conditional Akaike’s information criterion (cAIC) [26, 27].

## Results

### Growth in STL & mass over lactation

**Fig 1:**
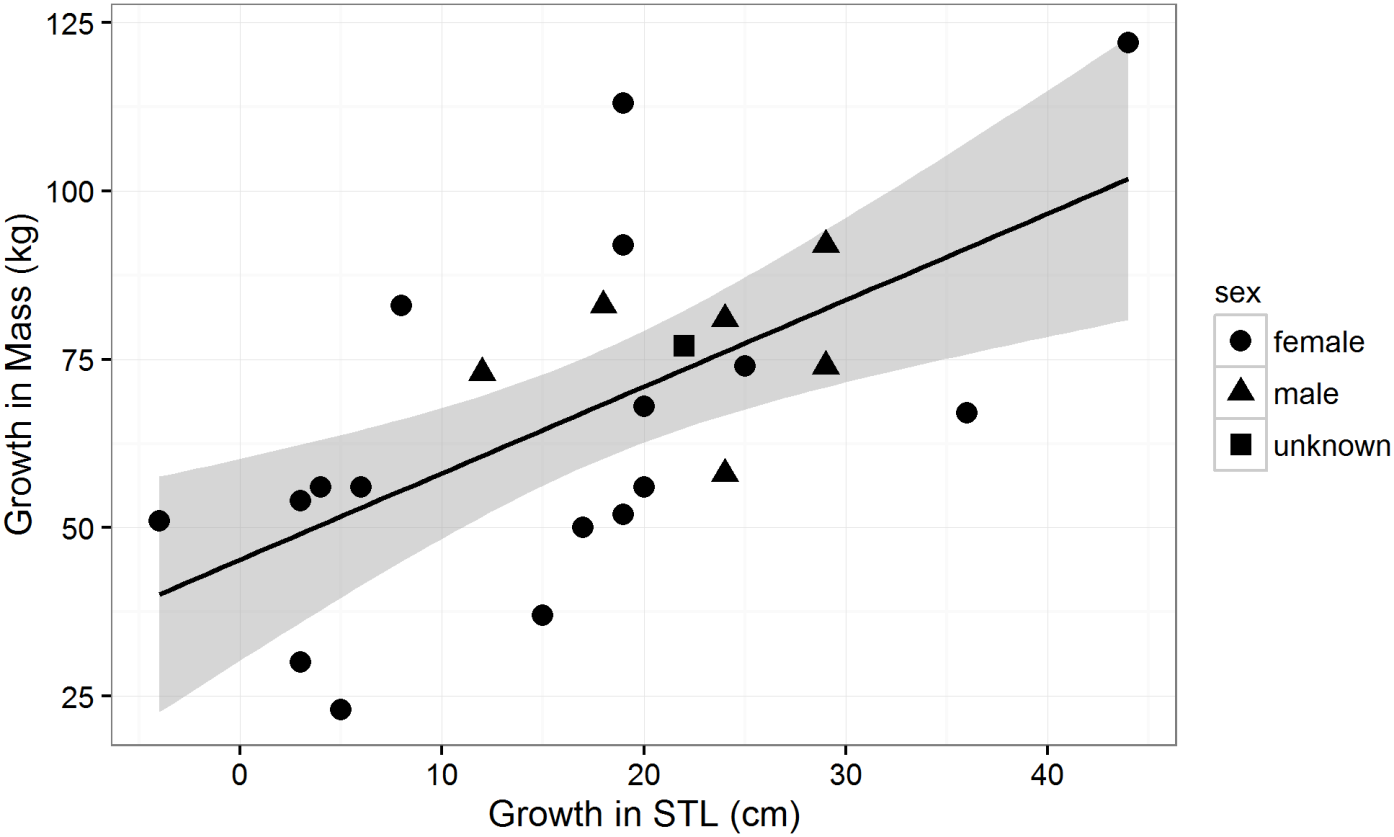
The relationship between growth in snout tail length (STL, *l*.*growth*) and mass (*m*.*growth*) in southern elephant seal pups at Macquarie Island over the 23 day lactation period. The regression equation was: *l*.*growth~ 45*.*25+1*.29* m.*growth*.

The mean STL and mass of the 24 pups sampled was 118 ± 9 cm and 42 ± 7 kg respectively at birth, and 135 ± 14 and 110 ± 27 kg at weaning, indicating clear growth in both components (Fig 1). Changes in both mass and STL over the lactation period were highly variable across the 24 seals studied, with changes in STL ranging from a maximum of 44cm to a minimum of -4cm, while changes in mass ranged from a maximum increase of 122kg to a minimum increase of 23kg. The mean increase in STL was 18 ± 11cm and the mean increase in mass was 68 ± 24 kg (Fig 1).

A significant positive relationship was found between *stl*.*growth* and *m*.*growth* over the lactation period, although this relationship was relatively weak (slope = 0.62; Fig 1). The results of the simple linear regression suggest that a significant proportion of the total variation in *m*.*growth* was predicted by *stl*.*growth* (F_(1,22)_ = 13.48, p < 0.05, adjusted R^2^ = 0.3517). According to this model, pup *m*.*growth* increased by 1.28 kg for every 1 cm increase in *stl*.*growth*. This relationship confirms that weaning STL can be used as a measure of maternal expenditure in southern elephant seals. Removal of seal that experienced much higher growth relative to other seals (increase of 44cm and 122kg, compared to the average across all samples of 17cm and 68kg) in the sample did not significantly change the relationship identified.

### STL at weaning

**Fig 2:**
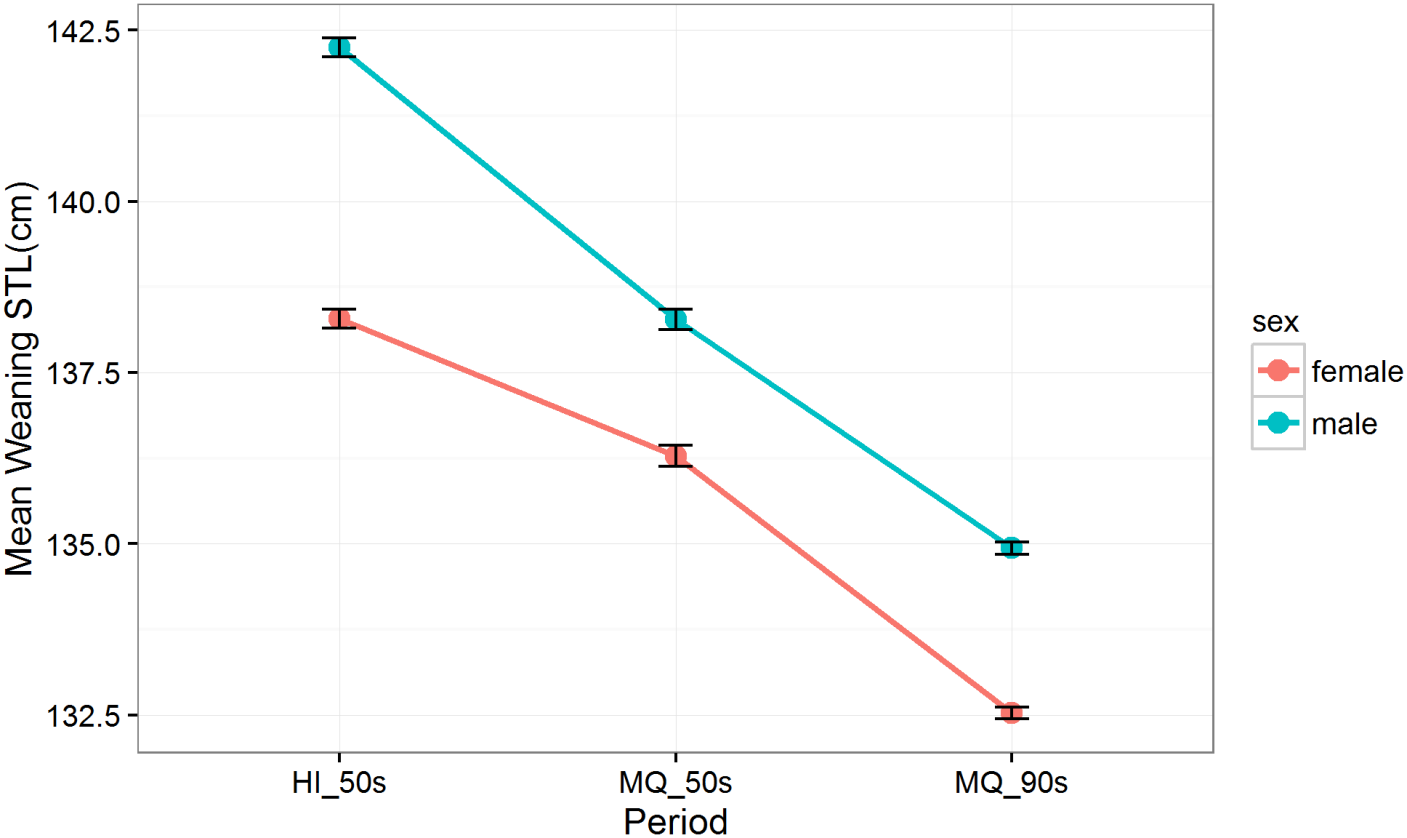
Estimated weaning *STL* (cm) for female (red) and male (blue) Macquarie Island Southern elephant seals between three study periods: Heard Island in the 1950s (HI_50s), Macquarie Island in the 1950s (MQ_50s) and Macquarie Island in the 1990s (MQ_90s). Estimates based on the linear mixed effects model: *STL ~ sex*:*period*, with year included as a random effect. 95% confidence intervals are indicated by the error bars.

The mean *STL* of the 8084 elephant seals at weaning varied between all three study periods (Fig 2). Males were, on average, 3 cm (a mean difference of 2.2%) longer than female weaners across the three periods, although the difference in the *STL* of males and females was most pronounced in the HI_50s period.

The top model explaining wean size in terms of *sex* and *period* (HI_50s, MQ_50s & MQ_90s) was include only the interaction between sex and period: Weaning *STL* ~ *sex*:*period* (ΔAIC between top and second model = 5.75, evidence ratio = 18; Table 1). Weaning STLs in female elephant seals were similar in the HI_50s and MQ_50s sampling periods. In contrast the lengths of males differed between island in the 1950s In males, weaning STLs were on average 3 cm shorter in both the 1990s (135±10 cm) than in the 1950s (138±12cm). Female pups had a wean STL of 133±11 cm in the 1990s compared to 136±11 in the 1950s, a mean difference of 3 cm.

**Table 1:** Model selection relating weaning size (snout-tail-length, STL) in southern elephant seals at Macquarie and Heard Islands (1949–2005), showing the conditional AIC (cAIC), the log Likelihood, delta cAIC, the model weighting (w) and the weighted cAIC (wcAIC). Note: period = (i) Heard Is: 1949–1953, (ii) Macquarie Island; 1954–1963 and (iii) Macquarie Island: 1987–2005.

Model	cAIC	logLikelihood	ΔcAIC	w	wcAIC
Weaning *STL* ~ sex:period	61072.59	-30513.9	0.00	1.00	0.91
Weaning *STL* ~sex	61078.34	-30518.6	5.75	0.06	0.05
Weaning *STL* ~ sex + period	61079.12	-30519.2	6.54	0.04	0.03
Null	61180.44	-30570.6	107.85	0.00	0.00
Weaning *STL* ~ period	61181.2	-30571.2	108.62	0.00	0.00

## Discussion

### Sources of error

There is potential for the temporal differences in weaning *STL* found in this study to be due to discrepancies in the sampling methods used between the 1950s and 1990s. During the 1950s, pups at Heard and Macquarie Island were principally measured by eye, by pacing or by drawing a line in the sand and measuring the distance [18], and thus the *STL* measurements from this period are likely to be less precise than those from the 1990s. While these less precise methods may result in a bias within certain years due to individual observers, it is unlikely that we would see a consistent bias across all 11 years and both sites from these study periods. The biggest problem associated with the greater uncertainty of the 1950s estimates is the reduction in the statistical power when comparing the two time periods. However, we were nonetheless able to identify differences in the weaning *STL* of pups in this study.

### Changes in STL & mass over lactation

There was a positive relationship between growth in mass and length during the lactation period, although this relationship was relatively weak, partly owing to our relatively small sample size. Nonetheless, the relative changes in *STL* and mass over the lactation period were similar to those reported by Bryden [23]where an increase in length over the 24 day lactation period was also documented, despite distinct differences in the methodologies used; Bryden [28] used a cross sectional approach, while the method used in this study was longitudinal.

The growth in pup length over the nursing period indicates that pups partition a proportion of their mothers’ energetic expenditure towards the development of somatic tissue (*i*.*e*., muscle and bones) and not put it all towards fat deposition to provide fuel for the up-coming fast. The dataset was, however, dominated by female seals, with too few males to detect sexual differences in growth during the lactation period. Differences in body composition at weaning between males and female pinniped pups have been reported in Antarctic, *Arctocephalus gazelle*, [29] and Australian fur seals, *Arctophoca pussilus*, [30], with males slightly heavier at weaning but having proportionately less body lipid reserves and more muscle than females, indicating greater somatic growth during the lactation period. Antarctic and Australian fur seals are extremely polygynous and sexually dimorphic, with the reproductive success of males strongly dependent on adult body size [29]. Sexual selection may, therefore, favour greater relative increases in somatic growth in male pups in order to gain an advantage in future male-male interactions [31]. Given that elephant seals are also highly polygynous and sexually dimorphic, we might expect to see similar sex differences in the body composition and partitioning of maternal resources. Any sex differences in growth are, however, unlikely to negate the relationship between changes in *STL* and mass found in this study, as males are likely to show greater growth in *STL* for every increase in mass, and thus more males in the data would only strengthen this relationship.

The increase in length during lactation also report by [32] indicates that the length of pups at weaning is likely to be influenced by maternal expenditure in a similar way to pup weaning mass. Consequently, weaning *STL* of elephant seal pups can be used as an index of maternal expenditure and foraging success and, more broadly, the relative state of the foraging conditions encountered by the female over the post-moult migrations. Further, because weaned pups lose a substantial proportion of their weaning mass over the post-moult fast [33–35], while generally maintaining weaning length [23], the *STL* of pups at weaning is, to some extent, a more robust measure of maternal foraging success compared to mass as it is not influenced by the time at which weaners are measured.

### Spatial differences in weaning STL during the 1950s

During the 1940-1960s, the mean *STL* of female elephant seal pups at weaning was similar at Heard and Macquarie Island, although in, contrast, male weaners during this period were significantly longer than their conspecifics at Macquarie Island. Elephant seal mothers tend to wean relatively larger male pups compared to females when foraging conditions in the winter are good [13, 36–38]. Therefore, the larger weaners at Heard Island during the 1950s suggests that the overall condition of mothers from Heard Island during this period was better than those from Macquarie Island. These differences might arise because of differences in the foraging behavior and habitat use between Heard and Macquarie Island mothers. Over the post-moult period, Macquarie Island females forage in either the pelagic waters of the sub-Antarctic or transit south over the deep, oceanic waters of the Antarctic Circumpolar Current (ACC) into the highly productive waters of the Antarctic continental shelf [39, 40]. Seals that transit south rarely forage *en-route* and consequently lose substantial body condition prior to reaching truly Antarctic waters [41]. While a proportion of the females from Heard and Kerguelen Islands transit south onto the Antarctic continental shelf like those from Macquarie Island, this transit is predominately across the preferred shelf habitat over the Kerguelen Plateau, and thus the Kerguelen Island females can forage opportunistically and gain body condition over the transit period [41]. Further, a proportion of the Kerguelen seals forage to the east of the Kerguelen Islands, where they track the highly productive Kerguelen Plume as it is advected to the east over winter [42, 43]. Therefore, the seals from the Kerguelen Islands that adopt a sub-Antarctic, frontal foraging strategy may do substantially better than those from Macquarie Island, where there is no such plume.

### Temporal changes in weaning STL

The mean *STL* of Macquarie Island elephant seal pups at weaning decreased by 3cm between the 1950s and 1990s, concurrent with the observed population decline. This finding supports the hypothesis that reductions in the foraging success of breeding females from Macquarie Island, in response to reductions in foraging conditions, are ultimately responsible for the population decline, as females allocate fewer resources to their pups, resulting in fewer surviving until breeding age. Even though this is a small change, the overall rate of decline for the Macquarie Island population of breeding females is less than 1% per annum. A very small but long-term change in first year survival (through a small change in weaning size) is therefore sufficient to generate this insidious rate of decline [38]. This also accords with a previous study at Marion Island which found that mean weaning mass was smaller than the long-term average during a period of population decline, with a sudden reversal in the growth rate of the population preceded by an increase in mean weaning mass [11].

This decline in weaning size stands in apparent contrast to a reduction in the mean age of primiparity among Macquarie Island seals from 5–6 years to 4 years of age reported over the same period [19, 22, 44]. The age of primiparity in female elephant seals is closely related to growth rate and, ultimately, foraging performance [45], with more successful foragers able to grow faster, become larger and breed earlier. A decrease in the age of primiparity therefore indicates a relative increase in the foraging success of sub-adult seals from Macquarie Island. We suggest that these contradictory findings can be explained by differences in the foraging conditions experienced by Macquarie Island seals during distinct ontogenetic phases of their life cycle. During the post-moult period, adult females from Macquarie Island forage in three distinct ocean realms: (i) the pelagic waters of the sub-Antarctic, (ii) the waters of the continental shelf and (iii) ice edge adjacent to the Victoria Land coast and the Ross Sea [3; 39, 40, 46]. In contrast, juvenile seals (under-yearlings to 3–4 years of age) predominately forage in the pelagic waters of the sub-Antarctic, typically avoiding Antarctic waters and the ice edge [47–50]. Therefore, changes in the foraging conditions in high latitude Antarctic waters (realms i and ii), for example as a result of the encroachment of sea ice and the exclusion of females from shelf waters [10, 39], are likely be reflected in the foraging success of the adult females using those regions and the size of their pups at weaning, rather than the growth rate of juveniles.

If conditions in the Antarctic foraging grounds depreciate, a proportion of females returning to Macquarie Island to breed will likely be in relatively poor condition, and will consequently wean pups in relatively poor condition. Despite being, to some extent, buffered by females that use the sub-Antarctic foraging strategy, the mean size of pups born and weaned at Macquarie Island will slowly decrease over time. Further, given the positive relationship between weaning mass and first year survival [24], mortality among under yearlings in years of poor Antarctic foraging conditions is likely to be relatively high, with only the largest and fittest pups surviving their first year [24]. The underlying physical mechanisms driving variation in food are complex. However, the Southern Annular Mode has been demonstrated to influence female foraging success at Macquarie Island, with mothers gaining less weight over the winter months when SAM is positive [51]. Further, SAM has been in a predominantly positive phase for the last three decades, so it may be an important proximal driver of population size.

Increasing mortality amongst under-yearlings would, however, result in reduced competition for, and therefore greater availability of, prey resources in the sub-Antarctic foraging grounds where juvenile seals forage. The relative increases in food availability would consequently result in relatively high growth rates among juvenile seals, enabling them to gain a suitable body size for breeding earlier in life and reducing the age of primiparity from 5–6 years to 4 years. The apparent slow growth rates (older age of primiparity) in the 1950s relative to the 1990s suggests that prior to population decline (*i*.*e*., the 1950s), the Macquarie Island population was likely to have been at, or close to, carrying capacity. Under these circumstances, prey resources would be limiting throughout their foraging range, including in the juvenile foraging grounds in the sub-Antarctic. As detailed above, the decline in the number of pups in the sub-Antarctic foraging grounds, driven by changing conditions in the Antarctic foraging grounds of adults, would have freed up resources, allowing juveniles greater access to prey and enabling more rapid growth rates and an earlier age of primiparity.
