# Correction: Five decades on: Use of historical weaning size data reveals that a decrease in maternal foraging success underpins the long-term decline in population of southern elephant seals (*Mirounga leonina*)

**DOI:** 10.1371/journal.pone.0195688

**Published:** 2018-04-04

**Authors:** The PLOS ONE Staff

## Notice of republication

This article was republished on March 21, 2018, to include the Abstract that was omitted during the typesetting process. The publisher apologizes for the errors. Please download this article again to view the correct version. The originally published, uncorrected article and the republished, corrected articles are provided here for reference.

## Supporting information

S1 File Originally published, uncorrected article. (PDF)

S2 File Republished, corrected article. (PDF)
